# Experimental investigation of a near-field focusing performance of the IP-Dip polymer based 2D and 3D Fresnel zone plate geometries fabricated using 3D laser lithography coated with hyperbolic dispersion surface layered metamaterial

**DOI:** 10.1515/nanoph-2023-0258

**Published:** 2023-07-24

**Authors:** Patrik Micek, Alexandr Belosludtsev, Tatjana Gric, Dusan Pudis, Peter Gaso, Matej Goraus

**Affiliations:** Department of Physics, Zilinska Univerzita V Ziline Katedra Fyziky, Zilina, Slovakia; Optical Coating Laboratory, Center for Physical Sciences and Technology, Vilnius, Lithuania; Department of Electronics, Vilniaus Gedimino Technikos Universitetas, Vilnius, Lithuania

**Keywords:** Fresnel zone plate, HMM, IP-Dip, nanoscribe, near-field probe, SNOM

## Abstract

Herein we investigate the character of the near-field emission of a two- (2D) and novel three-dimensional (3D) probe geometries fabricated using 3D direct laser writing lithography. Near-field scans in *x*–*y* and *y*–*z* planes were measured both before and after the deposition of hyperbolic dispersion metamaterial (HMM) to further verify the directional propagation of the high wave-vector components present in the vicinity of the structures. Additional computational and theoretical characterization forewent the actual experimental measurements, showing a promising performance, particularly for the 3D Fresnel zone plate (FZP). Overall, the experimental data documents a subwavelength resolution with a significant signal enhancement in the focal spot of the 3D FZP and highly subwavelength depth of focus.

## 1 Motivation

Subwavelength imaging has been the topic of interest for quite some time, fuelling the development of multiple microscopy techniques such as confocal microscopy, scanning electron microscopy (SEM) or scanning near-field optical microscopy (NSOM) [1–4]. The latter has consistently proven to be an immersive and highly-useful tool to optically characterize properties of samples at the nanoscale by carefully observing the sample-probe interaction during the scanning. The angular resolution of the NSOM relies purely on the optical probe used. Currently, the state-of-the-art scattering scanning near-field optical microscopes (s-SNOM) use metal coated silicon probes in contact mode in combination with continuous scanning motion to effectively collect scattered near-field light from the sample during the contact [5–8]. A non-contact scanning technique using a feedback shear force mechanism to precisely control the probe-sample distance developed before the s-SNOM takes advantage of aperture or aperture-less metal-coated optical fiber-based probe near-field coupling to create an image of the sample. Fabrication of such probes is linked with costly deposition and lithography techniques such as electron-beam physical vapor deposition (EB-PVD) or focused ion beam (FIB) lithography and the physical properties of the deep subwavelength aperture itself put a limit to the optical throughput of such a device [9, 10]. There are several types of aperture-less near-field probes being used in state-of-the-art systems for the optical characterization of samples with resolution reaching up to tens of nanometers [11–14]. Scattering probes are experiencing a noticeable growth in popularity due to their ability to be implemented into already existing atomic force microscopy (AFM) setups with precise scanning and feedback mechanisms, which allow for a detailed imaging at almost atomic scale [15, 16]. Although the obvious advantages, in order to obtain the scattered evanescent fields, the tip of the probe has to be in a close vicinity of the scatterer in order to obtain the near-field signal. To be precise – to obtain the best resolution, the probe has to maintain a physical contact during the measurement. Therefore, the probes used in s-SNOM configurations have to be replaced regularly. On the other hand, the aperture-less near-field probes based on tapered dielectric structures or pulled optical fibers offer a low-cost fabrication, currently being done on the facets of optical fibers by modern 3D lithography such as direct laser writing (DLW) or TPA lithography. The main issue of these probes is the evanescent decay of the propagating modes bellow the critical width of the tapered fiber. This resolves in an actual limit to the physical dimensions of the taper. From our previous work a planar structure consisting of a series of equidistant rings based on IP-Dip photoresist report a noticeable enhancement of the central peak in the near-field [17]. To further continue this work, we designed, manufactured and experimentally verified the effect of implementing a (HMM) on top of Fresnel zone plate (FZP) structures. Mote et al. experimentally demonstrated a functional phase FZP fabricated by FIB achieving resolution well below Abbe diffraction limit supported by multiple previous theoretical studies [18–20]. Recent advances in artificial materials might have unlocked the ability for the low-cost aperture-less probes to compete with the previous mentioned in terms of resolution by taking advantage of hyperbolic dispersion of layered metamaterials [21, 22]. By engineering the electric permittivity and magnetic permeability of a material, it is possible to obtain a unique optical response especially in the case of high wavevector fields such as evanescent waves. In this paper we present a 2D and 3D FZP near-field probe fabricated by 3D two-photon absorption (TPA) lithography as well as a numerical characterization and experimental verification of the fabricated device focusing performance. Additionally, we implemented an HMM metamaterial on to these probes to further enhance their near-field response.

## 2 Design

**Figure 1:**
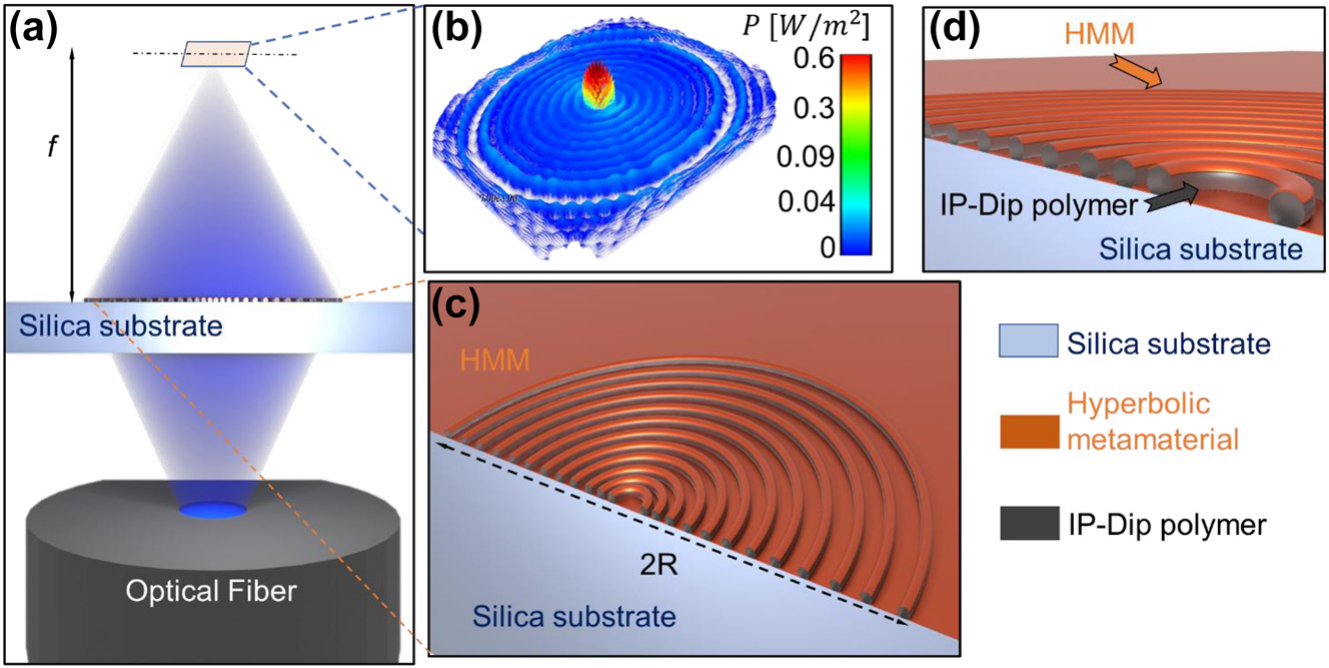
Schematics of the 2D Fresnel zone plate design with its (a) working principle, where the incident 420 nm light is transmitted from the multimode optical fiber through the Silica substrate onto the structure, which modulates the phase of the transmitted wave resulting in a strong enhanced signal at the focal plane, (b) a simulation of the Poynting vector amplitude distribution at the focal plane of the 2D Fresnel zone plane antenna, (c) schematic of the structure physical parameters, with 2R depicting the diameter of the FZP antenna, (d) detail of the schematic with each optical layer distinguished. The image is not to scale.

The overall conceptualization is based on the FZP design, where each zone influences the phase of the transmitted wave so that they interfere constructively at a given distance of the focal plane. The main input parameters characterizing an FZP are its diameter, the focal length, the refractive index of the structure and the incident wavelength [23–25]. These parameters directly influence the radius of each zone according to the equation

$$r_{k}=\;\sqrt{\frac{k\lambda_{0}}{n}f+\frac{k^{2}{\lambda_{0}}^{2}}{4n^{2}}},$$

where *n* is the refractive index contrast of the FZP material, *λ*_0_ is the incident wavelength in vacuum, *f* is the focal length of the FZP and *k* is an integer number associated with each Fresnel zone of the FZP. It is worth mentioning, that in this paper the aim was to investigate the near-field emission of the FZP and its potential usage as a terahertz antenna for near-field imaging, therefore for the design part, the second part of the equation under the square root cannot be neglected as is commonly done for Fresnel lenses designed to focus light to an object placed at infinity. The implying structure was designed for *λ*_0_ = 420 nm, the focal length of 300 nm and refractive index of the IP-Dip polymer *n* = 1.53. The total number of the FZP zones was chosen to be 11, in order to address the previously documented results of the efficiency gain of FZPs with the higher number of zones. The overall design as well as a simulation of the in-plane near-field emission at the focal distance is shown in Figure 1. The diameter of the whole structure is 12.15 μm, indicated in Figure 1(c) as the distance 2R, while the height of each zone is 760 nm with the HMM included. The zone axial parameters rely mainly on the size of the voxel of the nanoscribe TPA system yielding an axial resolution of 300 nm while the height of the voxel reaches up to 500 nm. This however can be dealt with by reducing the laser power, increasing the scanning speed or by focusing the voxel slightly below the glass/IP-Dip interface so that the overall dimensions exceed the considered resolution limits of the lithography.

**Figure 2:**
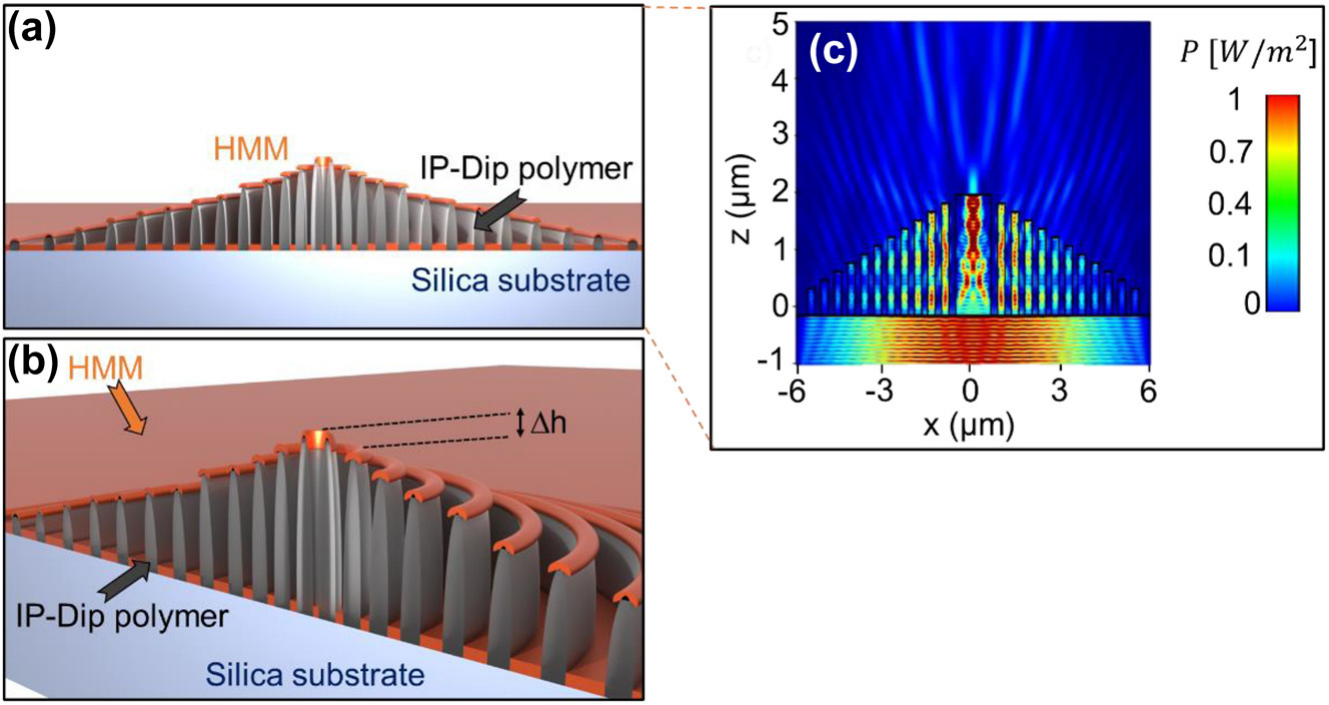
Schematic of the 3D FZP probe as can be viewed from the (a) *x*–*z* plane, (b) perspective detail with the step-like height function of each zone highlighted, (c) cross-section of the FDTD simulation of the Poynting vector amplitude.

Additionally to the planar geometry of the FZP, a 3D equivalent of the planar design was designed and fabricated, with the step-like variation of the height of each zone. We introduced a linear height increasement from the outside of the structure towards its center as indicated in Figure 2(b). The overall structure is depicted in Figure 2(a). The aim was to compare the overall near-field probe efficiency and signal enhancement as well as the achieved resolution. The overall radius of the device as well as the radius of individual zones remained, however, the number of zones increased to 12 so that the tallest innermost zone is included in the structure. This would prove to be an effective way to suppress the side lobes emerging from the spacers separating the Fresnel zones and thus resulting in an improvement of the probe signal enhancement and axial and lateral resolution improvement.

To better understand the effect of the focusing behavior of the deposited HMM, the near-field of both the 2D and 3D structure design was characterized before the actual deposition of the metamaterial. Both 2D and 3D geometries shown in Figures 1 and 2 were simulated using commercially available Ansys Lumerical 2021. The size of the simulation region was set up to 12 μm × 12 μm × 6 μm with an override mesh region of 1 nm^3^ overlaying each zone completely. All the boundary conditions were set up to a perfectly matched layer (PML). Here it is worth mentioning, that its possible take advantage of the structure symmetry by evaluating the boundary conditions to symmetric and antisymmetric.

### 2.1 Design and simulation of the layered HMM

**Figure 3:**
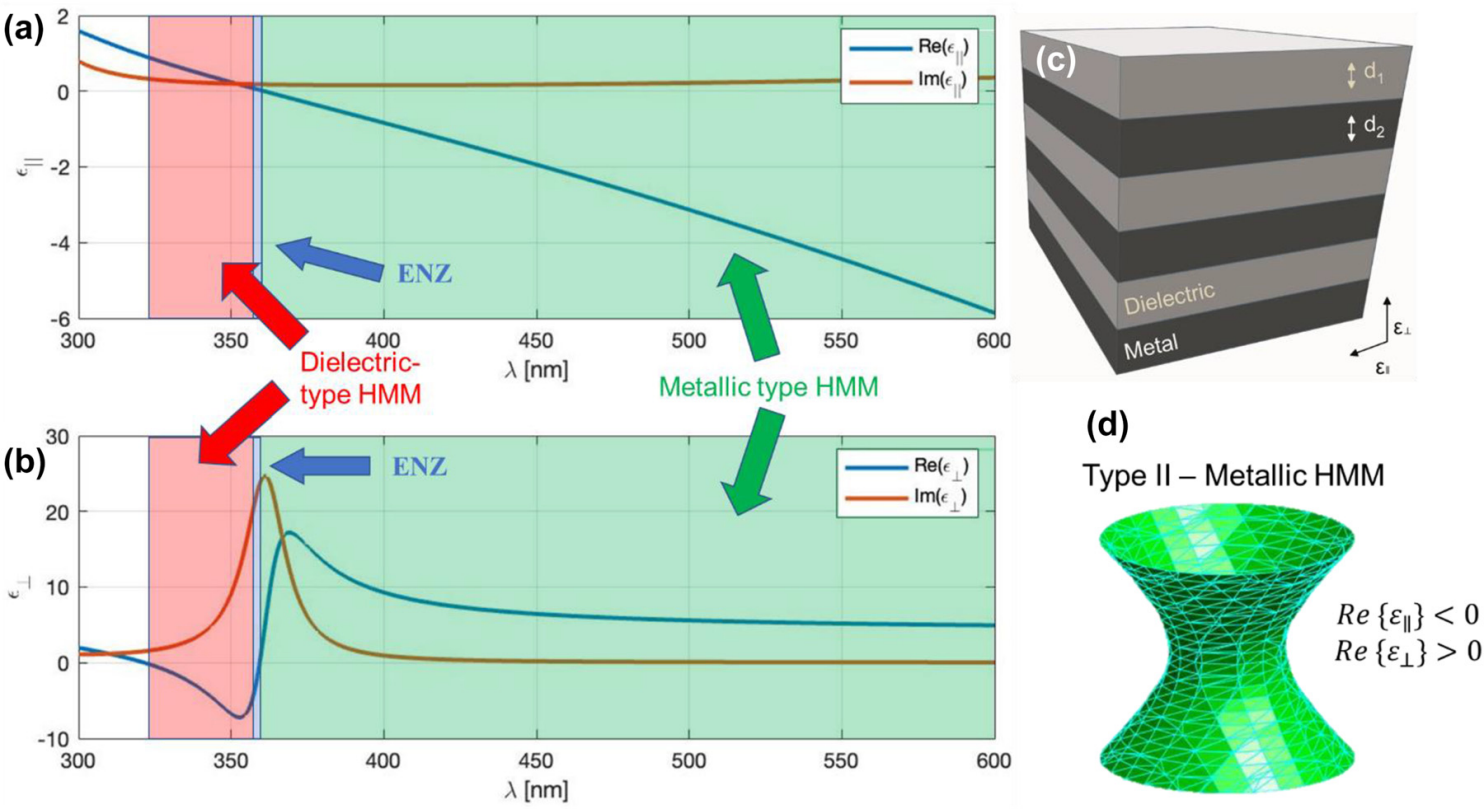
Characterization of the spectral dependence of the (a) parallel and (b) permittivity tensor components of the deposited metamaterial consisting of *x* pairs of periodical layers of silver and SiO_2_ with thicknesses of 13 nm and 10 nm respectively. (c) Detail schematic of the layered metamaterial. (d) Resulting IFC depicted from the metallic type bandwidth regime depicted from (a) highlighted in green color.

The goal of achieving a deep-subwavelength resolution of an aperture near-field probe can also be addressed by specifically designing the dispersive material at the exit plane behind the apex of the probe which forces a highly directional propagation of the high-wavevector evanescent fields. In convectional isotropic non-dispersive media, the dispersion of the medium is characterized by an equation

$$\left(\frac{k_{x}^{2}+k_{y}^{2}+k_{z}^{2}}{\varepsilon }\right)=k^{2},$$

where *k*_*x*_, *k*_*y*_ and *k*_*z*_ are the components of the wavevector in the isotropic medium along the corresponding axis, respectively, *ε* is the permittivity of the isotropic medium and ***k*** is the wavevector. The isotropy of the permittivity tensor components creates a spherical isofrequency contour (IFC), where the group velocity vector matches the allowed wave-vector of the surface, leads to the photonic density of states (PDOS). One can directly influence the IFC of the medium by incorporating nanostructures with dimensions much smaller than the wavelength of the incident electromagnetic radiation, thus creating an effective response of the material. These artificially created composites called metamaterials exhibit numerous unnatural dispersion properties due to their collective response resulting from the interaction of each metamaterial layer. An example of a metamaterial is a bulk media consisting of periodically arranged thin layers as shown in Figure 3 below.

Such a metamaterial exhibits a highly anisotropic permittivity tensor which due to the dispersive character of the metal layers effectively tunes the IFC of the material leading to a two-fold or one-fold hyperboloid. The IFC of a hyperboloid results from the fact, that one of the components of the permittivity tensor has a negative value, which effects the studied hyperbolic metamaterial as an anisotropic media. In that case, the permittivity tensor takes the form as follows

$$\frac{k_{x}^{2}+k_{y}^{2}}{-\varepsilon_{\|}}+\frac{k_{z}^{2}}{\varepsilon_{⊥}}={\left(\frac{\omega }{c}\right)}^{2},$$

where *ω* is the angular frequency, *c* is the speed of the light in vacuum, *ε*‖ and *ε*⊥ is the parallel and perpendicular component of the permittivity tensor for the *x*–*y* plane of a layered hyperbolic metamaterial, respectively, given by Maxwell–Garnet effective media approach as follow

$$\varepsilon_{\|}\left(\omega \right)=\;\rho \varepsilon_{\text{m}}+\left(1-\rho \right)\varepsilon_{\text{d}},$$

$$\varepsilon_{⊥}\left(\omega \right)=\;\frac{\varepsilon_{\text{m}}\varepsilon_{\text{d}}}{\rho \varepsilon_{\text{d}}+\left(1-\rho \right)\varepsilon_{\text{m}}},$$

where *ε*_d_ is the permittivity of the dielectric layer, *ε*_m_ is the complex permittivity of the metal layer and *ρ* is the aspect ratio given by *ρ = d*1*/d*2, where *d*1*,*2 are the thicknesses of the dielectric and metal layer, respectively. It is worth mentioning that in isotropic materials, light can propagate in all in-plane directions, however, in the HMM IFC directly influences the propagation of electromagnetic fields in preferred direction. It is also worth mentioning that if all the components of the relative permittivity tensor are positive, either an isotropic dielectric medium (IFC has a surface of a sphere) or an anisotropic dielectric medium (IFC has a surface of ellipsoid) is obtained. On the other hand, if all the components are negative, a bulk metal media response is obtained. The width of the layers has to be sufficiently small in order to apply the effective medium approximation and avoid photonic crystal-like behavior, where diffraction is the dominant mechanism determining the bandwidths of propagating optical modes. If the components of the relative permittivity tensor an opposite in signs as follow *ε*_‖_ > 0, *ε*_⊥_ < 0, the resulting IFC takes the form of the two-fold hyperboloid shown in Figure 3(d). In the other case when *ε*_‖_ < 0, *ε*_⊥_ > 0, the IFC takes a shape of a one-fold hyperboloid. The IFC directly influences the dipole source emission in a given material which radiation pattern can be obtained by Green’s function analysis. Silver with frequency-dependent relative permittivity given by the Drude–Lorentz dispersion model

$$\varepsilon_{\text{Ag}}\left(\omega \right)=\;1-\frac{\omega_{\text{P}}^{2}}{\omega \left(\omega +i\Gamma \right)},$$

where *ω*p is the bulk plasma frequency of silver, *ω* is the angular frequency and Γ term is the electron collision frequency was chosen for the metal layer. The angular plasma frequency and electron collision frequencies of silver were chosen to be *ω*p = 2.321 × 1015 rad/s and Γ = 5.513 × 1012 rad/s, respectively. For the dielectric layer, SiO_2_ with relative permittivity of 2.15 was chosen.

## 3 Experimental

**Figure 4:**
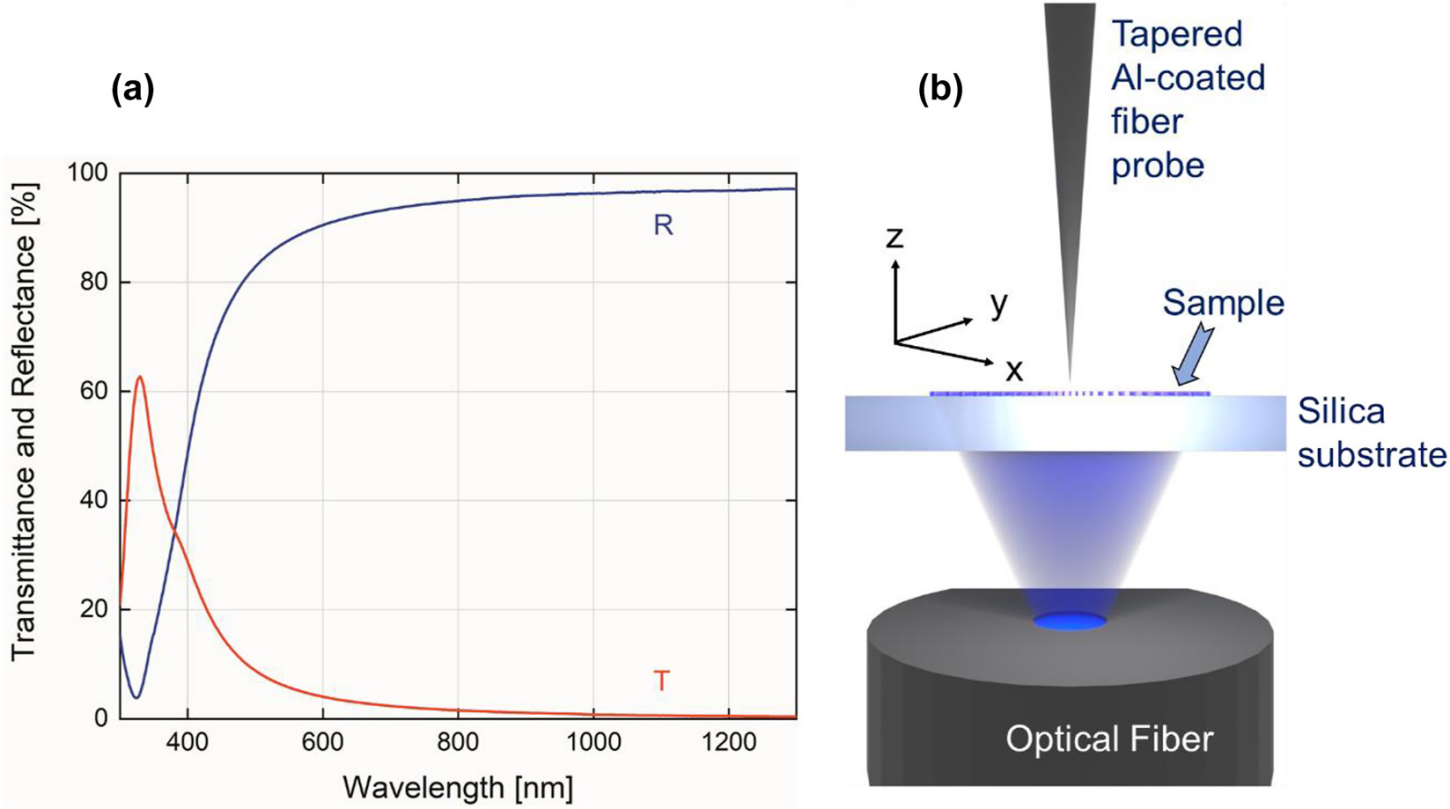
Experimental measurements of the (a) reflectance and transmittance of the deposited Ag/SiO_2_ HMM and (b) schematic of the NSOM measurement setup in collection mode.

**Figure 5:**
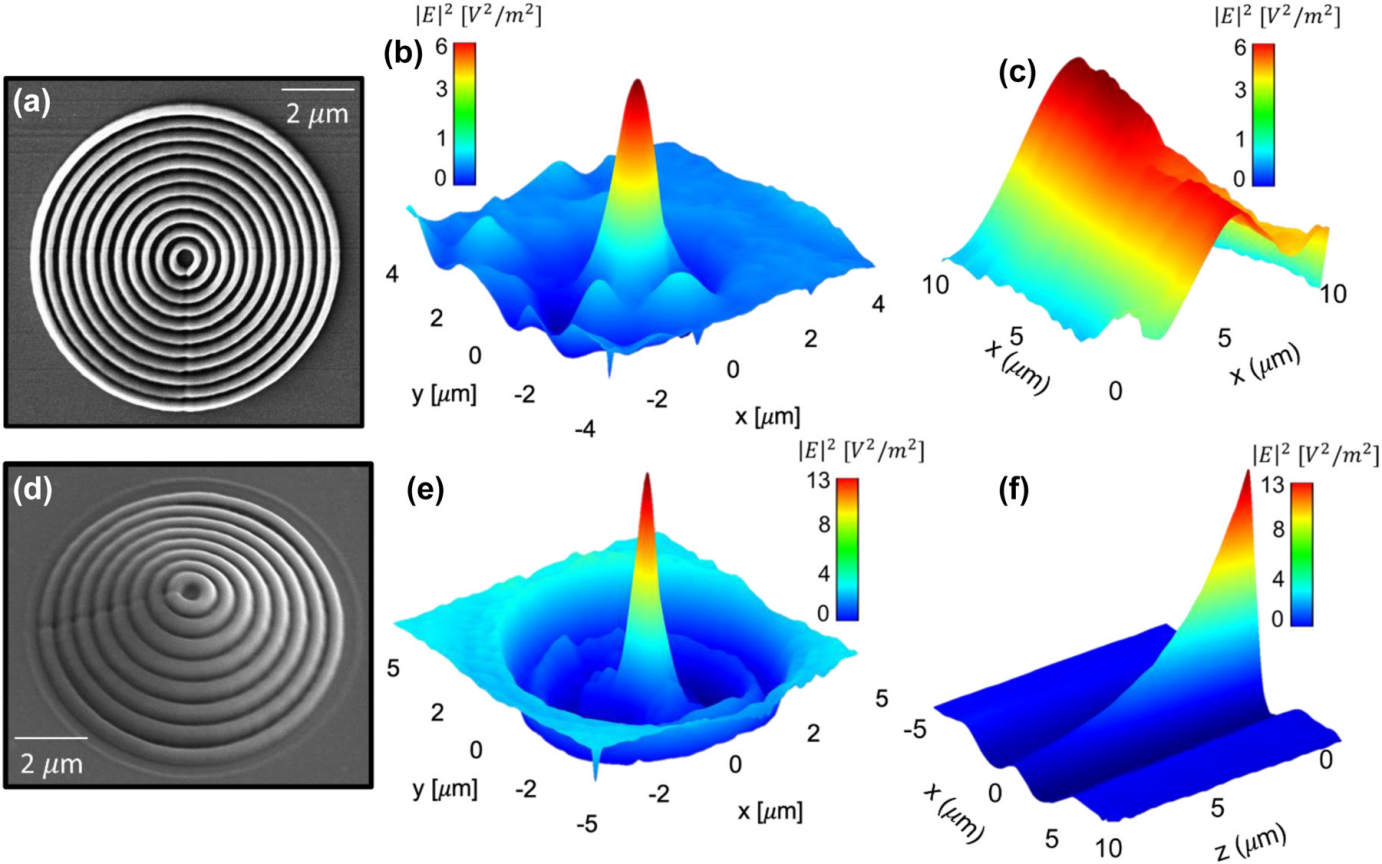
Experimental characterization of IP-Dip, (a) 2D FZP topography, (b) *x*–*y* plane NSOM measurement of the 2D FZP, (c) DOF NSOM measurement of the 2D FZP, (d) 3D FZP topography at tilted view of 30°, (e) *x*–*y* plane NSOM measurement of the 3D FZP and (f) DOF NSOM measurement of the 3D FZP.

**Figure 6:**
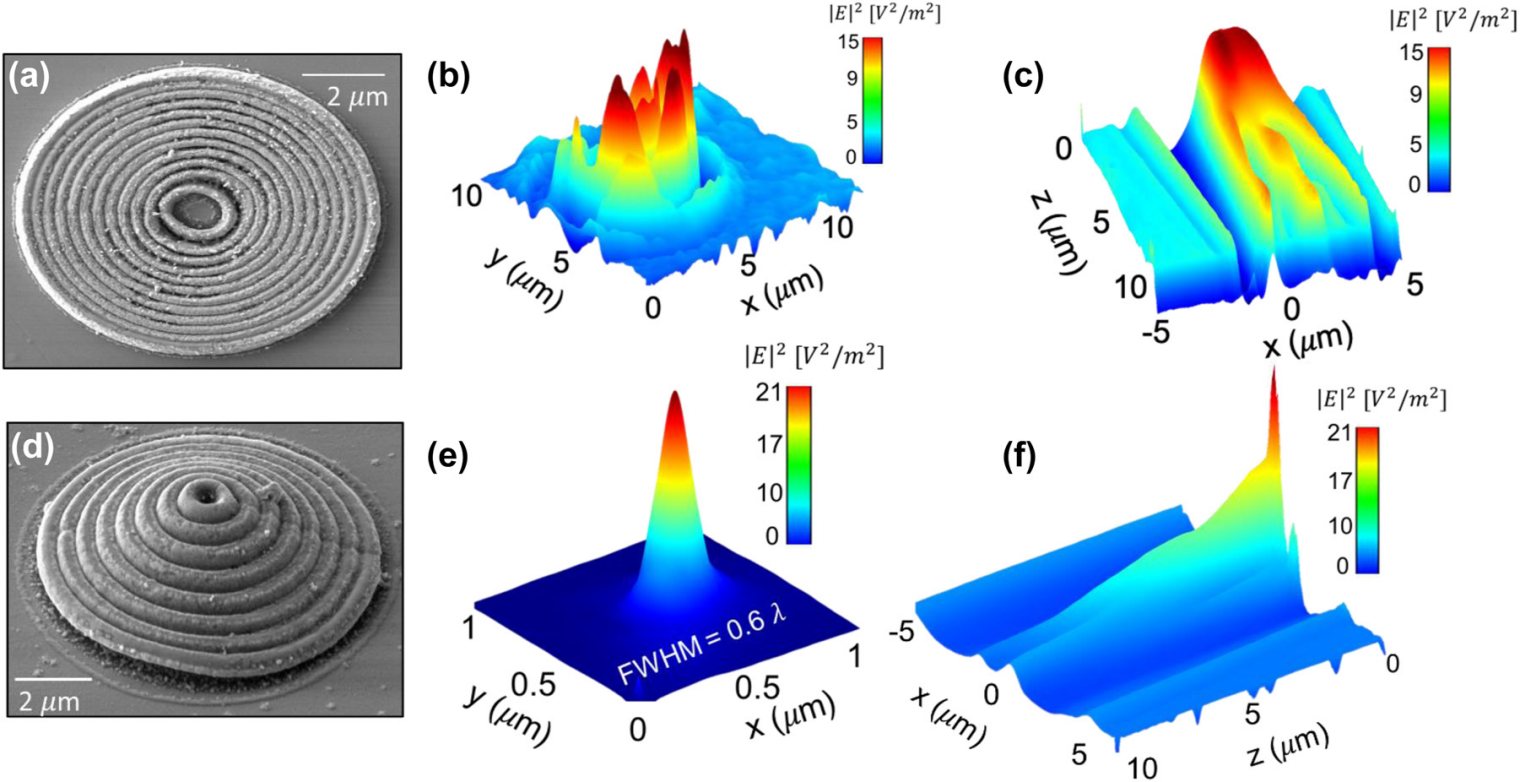
Experimental characterization of HMM deposited, (a) 2D FZP topography, (b) *x*–*y* plane NSOM measurement of the 2D FZP, (c) DOF NSOM measurement of the 2D FZP, (d) 3D FZP topography at tilted view of 30°, (e) *x*–*y* plane NSOM measurement of the 3D FZP and (f) DOF NSOM measurement of the 3D FZP.

**Figure 7:**
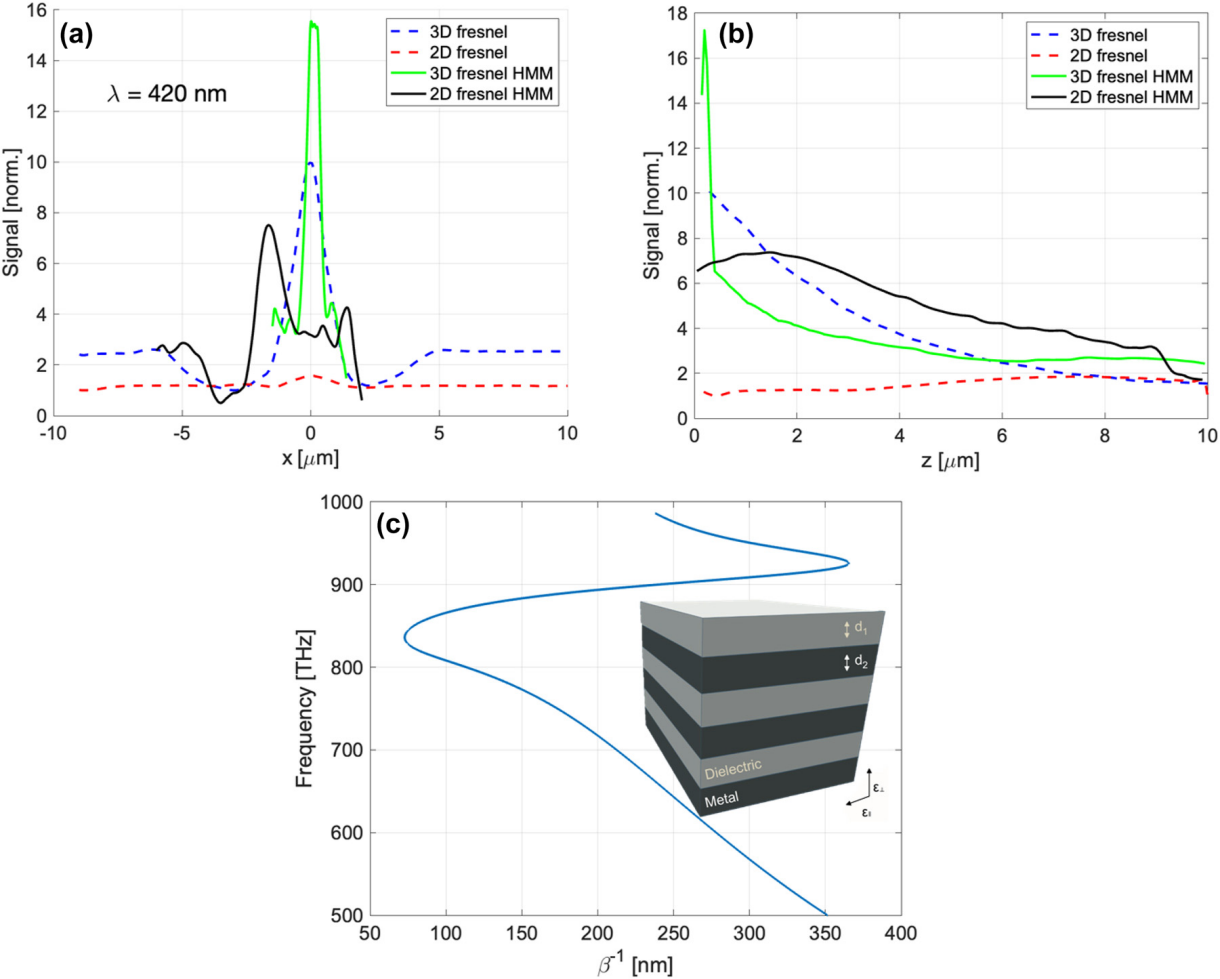
Experimental data extracted from the NSOM scans depicting, (a) cross-section of the focal plane (*x*–*y* plane) NSOM measurement both for the 2D and 3D FZP probes before (dashed lines) and after (full lines) the HMM deposition, (b) DOF of the fabricated probes extracted from the Figure 5(c) and (f) before (dashed lines) and after (full lines) the HMM deposition, (c) detail of the DOF in the vicinity of the FZP.

Non-linear process of the two-photon polymerization (TPP) based on the simultaneous absorption of two photons known as TPA was used for the fabrication of the IP-Dip photoresist-based FZP structure, where photo-initiators activated during the absorption form radicals which are suitable for the final polymerization in a small focal volume of the objective. As it is a non-linear process, high intensities of the incident light are required. This condition is only met within a small diffraction-limited area of the focus of the objective lens called voxel which reaches a spatial resolution of several hundred nanometers. For the fabrication, a commercial direct laser writing (DLW) system from nanoscribe GmbH (Karlsruhe, Germany) with a built-in femtosecond laser source with the wavelength of 780 nm and a pulse duration of 100 fs was used. Dip-in Laser Lithography (DiLL) configuration, where the 63× magnification and 1.4 numerical aperture microscope objective was submerged directly into the IP-Dip photoresist deposited at the glass silica substrate, was used in order to avoid spherical aberrations. The achieved resolution of this configuration reaches up to 250 nm depending on the scanning speed and laser power output of the femtosecond laser. For the quasi-planar FZP structure the parameters laser power and scanning speed were chosen to be 14 mW and 10,000 μm/s, respectively, in order to address the local central defect formation due to an increased polymerization reported in our last study. A sample was developed in the propylene glycol methyl ether acetate (PGMEA) for 5 min and submerged in isopropyl alcohol for 4 min. Figure 4 documents the experimental results of the reflectance and transmittance measurement of the layered HMM as well as the overall geometry of the NSOM measurement setup. Collection NSOM mode of transmitted light with a heat-pulled aluminium-coated optical fiber probe was used as the main tool for the near-field characterization of the structure. For the NSOM setup arrangement, the light emitting diode (LED) source was coupled into the FG105LCA multimode optical fiber with the numerical aperture of 0.22 and the core diameter of 105 μm and the fiber illuminated the structure from the silica substrate side. The LED with central emission at 420 nm was chosen. The HMM consisting of 6 pairs of Ag/SiO_2_ layers with the thickness of 13 and 10 nm (Figure 3(c)), respectively have been deposited on the IP-Dip samples using magnetron sputtering system PVD 225 (Kurt J. Lesker, Jefferson Hills, PA, USA). After the deposition, the transmittance (T) and reflectance (R) spectra of the deposited coating structure were measured. It is clearly seen the high R values from about 500 nm which is typical for silver. For silver, deposition conditions and precise thickness film control are described in [26]. Previous, optimization silicon oxide deposition took place as reported in [27, 28]. Direct transmittance and specular reflectance spectra of the manufactured films were measured in the 300–1300 nm wavelength range using a photon RT (EssentOptics Europe, Lithuania, Vilnius) spectrophotometer operating at normal incidence in transmittance and at the angle of 8° in reflectance mode. For these measurements, the sample deposited on a 1 mm thick double-side polished fused silica substrates was used. Figure 5 documents the SEM sample topography measurement and the NSOM scans of the fabricated structures at the focal plane (*x*–*y* plane) as well as their depth of focus (DOF) measurement for both 2D and 3D geometries before the deposition of HMM. Results show a good match comparing the designs to the overall angular resolution. Weak structure defect in 3D FZP is caused by the nature of the fabrication process as the starting/ending point creates slight discontinuity. The overall diameters of the structures is very close the design of 12.15 μm. After the deposition of the HMM, SEM images show the presence of the small nanoparticles located at the edges of the individual rings visible in Figure 6(a) and (d). It is also worth mentioning, that the key feature of the etched spacer in between the rings of the FZP cannot be clearly distinguished primarily in the case of the 3D design. However, the chosen fabrication algorithm proved to be an effective way to effectively form a small aperture (Figure 5(d)) with the diameter of 320 nm. Comparing the results for the two geometries, from Figure 5, the fabricated 3D FZP probe documented an exemplary result even before the actual HMM deposition, which is a great reference to the investigated self-focusing and near-field enhancement characterization of the HMM. The Fresnel diffraction emerging within the vicinity of the exit plane of the 2D FZP, forms a series of spherical inner zones with the additional annular concentric rings. As the distance between the capturing NSOM probe and the exit plane of the fabricated sample is decreasing the character of the propagating wave near the structure becomes spherical instead of plane-wave. Figure 6(b). The rings were present with all the FZP structures consequently although the emission strength across the probe varied. There are several factors responsible for this behavior. First, the fabrication process itself causes the photoresist to polymerize non-symmetrically, creating a region of attenuated height clearly visible in Figure 5(a) and (d) at the right-side of the 3D FZP probe. This disturbance in the structure height results in a different phase change of the transmitted light affecting the formation of the focal spot well documented for especially quasi-planar Fresnel zone plates. The 3D DLW photolithography was accompanied with undesirable polymerization of elevated volume of IP-Dip due to the fact, that the DLW process is a scanning technique indicating that the 3D designs were fabricated layer by layer. The spacers separating the rings measure down to 200 nm, reaching the spatial resolution limit of the nanoscribe. In combination with the character of the fabrication in the case of the 3D probes, the spacers are more likely to undergo polymerization. Nonetheless, the achieved signal enhancement with respect to the probe background averages near the factor of 16 for the 3D FZP probe accompanied with the measured resolution of *λ*/1.4 reaching subwavelength bandwidth documented in Figure 6(e) and (f) as well as in Figure 7(a) as the highly spatially confined narrow peak both in the *x*–*y* and *x*–*z* planes (green lines). Overall, the 3D design was expected to outstand, since its shape and size resemble that of a tapered cylinder or a cone. The near-field emission therefore can be well-explained by a guided mode theory, since the width of the innermost cylinder does not fulfill the critical waist size meaning that there are side peaks present in the captured data. Additionally, to the lateral NSOM maps, we investigated the depth of the field and the spatial resolution of each FZP design after the HMM deposition documented in Figure 6(c) and (f). As mentioned earlier, the HMM allows the propagation of the high-wave vector fields theoretically without attenuation, meaning that the side lobes caused by Fresnel diffraction are expected to dominate the near-field emission, which is the case for the 2D design, where the Fresnel diffraction is not attenuated. The effect of the near-field enhancement is clearly documented in the DOF measurement depicted in Figure 7(a) and (b). As the probe scans the *z*-direction at position of *x* = 0 the optical signal drops rapidly within 200 nm away from the central zone of the 3D FZP. Such an exponential decay is associated with strongly confined evanescent waves as a result of the imaginary nature of the wave-vector. For the wavelength of 420 nm, corresponding to the frequency of 713 THz, the propagation distance in the perpendicular direction from the HMM interface averages around 200 nm, which is in fair agreement with the simulated propagation distance shown in Figure 7(c). Clear enhancement of the evanescent waves directly above the probes was documented resulting in a strengthened optical signal near the probe surface. Figure 7 shows the results of these near-field characterizations. For the 2D FZP design, the localized field enhancement shows clear behavior of the propagation along the resonant cone of the IFC as was initially planned, however, due to the nature of the planar design, the emission width turned out not to be narrow. The 3D FZP NSOM maps (Figure 6(e) and (f)) document a subwavelength narrow peak emission with immediate evanescent decay along the surface normal above the probe. The resulted FWHM measured *λ*/1.4 in the spatial coordinates, which was close to the resolution limit of the capturing probe. Compared to the 3D FZP probe before the HMM deposition (Figure 7(a) blue dotted line) the resolution improved by roughly 300 %. The HMM shows a promising optical signal enhancement of the probes documented in both Figures 6 and 7. The 3D FZP takes advantage of the HMM thanks to the enhancement of the propagation distance of the large wavevector components, which in combination with the taper-like geometry causes the evanescent modes excited at the HMM-air interface to propagate along the interface up to the apex where they re-radiate to the free space. This way, the resolution of the probe is able to reach the subwavelength character.

## 4 Conclusions

It was demonstrated both theoretically and experimentally the effect of the directional propagation of the high wave-vector components propagating through the 2D and 3D FZP probes fabricated by 3D DLW photolithography based on polymer basis. The results show a narrow directional propagation with a detected signal enhancement reaching a factor of 16, a spatial resolution of *λ*/1.4 and unprecedented DOF below 200 nm. These promising results are crucial for further probe development and further implementation of the probe to an optical fiber leading up to an evaluation of its future application possibilities in NSOM. Additionally, the results showed that by designing an algorithm by which the structures were manufactured, it is possible to end up with a structure strongly resembling that of a conventional pulled optical fiber with an aperture already formed. The diameter of the aperture as well as its shape is strictly dependent on the optical power of the laser as well as on the scanning speed of the lithography.
